# Climate Change Drives the Transmission and Spread of Vector-Borne Diseases: An Ecological Perspective

**DOI:** 10.3390/biology11111628

**Published:** 2022-11-07

**Authors:** Jian Ma, Yongman Guo, Jing Gao, Hanxing Tang, Keqiang Xu, Qiyong Liu, Lei Xu

**Affiliations:** 1 Vanke School of Public Health, Tsinghua University, Beijing 100084, China; 2 Institute for Healthy China, Tsinghua University, Beijing 100084, China; 3 Respiratory Medicine Unit, Department of Medicine & Centre for Molecular Medicine, Karolinska Institutet, 171 77 Stockholm, Sweden; 4 Clinical Pharmacy Center, Nanfang Hospital, Southern Medical University, Guangzhou 510515, China; 5 State Key Laboratory of Infectious Diseases Prevention and Control, National Institute for Communicable Disease Control and Prevention, Chinese Center for Disease Control and Prevention, Beijing 102206, China

**Keywords:** climate change, vector-borne diseases, transmission, spread, interaction, COVID-19 pandemic

## Abstract

**Simple Summary:**

Vector-borne diseases (VBDs) are a major threat to human health. Climate change has a significant impact on VBDs. To clarify the complex effects of climate change on VBDs, we concluded the effects of climate on the transmission and spread of VBDs from an ecological perspective and summarized VBD changes in response to climate change, specifically including: the nonlinear effects of local climate (temperature, precipitation and wind) on VBD transmission, especially temperature showing n-shape effects; regional climate (the El Niño–Southern Oscillation and North Atlantic Oscillation) has time-lag effects on VBD transmission through indirect impact on local climate; and the u-shaped effect of extreme climates can lead to the geographical spread of VBDs. In terms of non-climatic factors, land use and human mobility through the interactions with climatic factors, will affect transmission and spread of VBD. We further explored the uncertainty of the impact of climate change on VBDs under the COVID-19 pandemic. A systematic understanding of the impact of climate change on the transmission and spread of VBD can provide insights and suggestions for future research on VBD prevention and control.

**Abstract:**

Climate change affects ecosystems and human health in multiple dimensions. With the acceleration of climate change, climate-sensitive vector-borne diseases (VBDs) pose an increasing threat to public health. This paper summaries 10 publications on the impacts of climate change on ecosystems and human health; then it synthesizes the other existing literature to more broadly explain how climate change drives the transmission and spread of VBDs through an ecological perspective. We highlight the multi-dimensional nature of climate change, its interaction with other factors, and the impact of the COVID-19 pandemic on transmission and spread of VBDs, specifically including: (1) the generally nonlinear relationship of local climate (temperature, precipitation and wind) and VBD transmission, with temperature especially exhibiting an n-shape relation; (2) the time-lagged effect of regional climate phenomena (the El Niño–Southern Oscillation and North Atlantic Oscillation) on VBD transmission; (3) the u-shaped effect of extreme climate (heat waves, cold waves, floods, and droughts) on VBD spread; (4) how interactions between non-climatic (land use and human mobility) and climatic factors increase VBD transmission and spread; and (5) that the impact of the COVID-19 pandemic on climate change is debatable, and its impact on VBDs remains uncertain. By exploring the influence of climate change and non-climatic factors on VBD transmission and spread, this paper provides scientific understanding and guidance for their effective prevention and control.

## 1. Introduction

Climate change, which has affected the world since the last century, has caused a general rise in temperatures over the period of 1906–2005 [1]. According to the Sixth Assessment Report (AR 6) of the Intergovernmental Panel on Climate Change (IPCC), the global average surface temperature will reach or exceed 1.5 °C in the next two decades, accompanied by increasing precipitation, melting glaciers and rising sea levels [2]. With the acceleration of climate change, extreme weather conditions will be frequent [3] and pose serious threats to human life and health. At present, about 30% of the global population is exposed to extreme weather that exceeds human thermoregulatory capacity for at least 20 days a year [4]. Moreover, global warming and extreme precipitation can contribute to the prevalence and expansion of diseases, leading to at least 150,000 deaths per year worldwide [5].

The ten publications in this Special Issue illuminate the impacts of climate change on ecosystems and human health from different perspectives in diverse disciplines, including phytology, biology, epidemiology, pathology, and molecular biology. In phytology and biology, the geographical ranges of plants and animals have been shown to be affected by climate change [6,7,8]. In epidemiology and pathology, the future geographic expansion of vector species that carry vector-borne diseases (VBDs) has been evaluated [9,10,11,12], and proved the invasive and evolutionary adaptation of vectors to different ecological and environmental conditions [13]. The impact of non-climatic factors on VBDs has also been assessed [14]. On the basis of these ten articles, we present a further discussion on climate change and VBDs.

**Figure 1:**
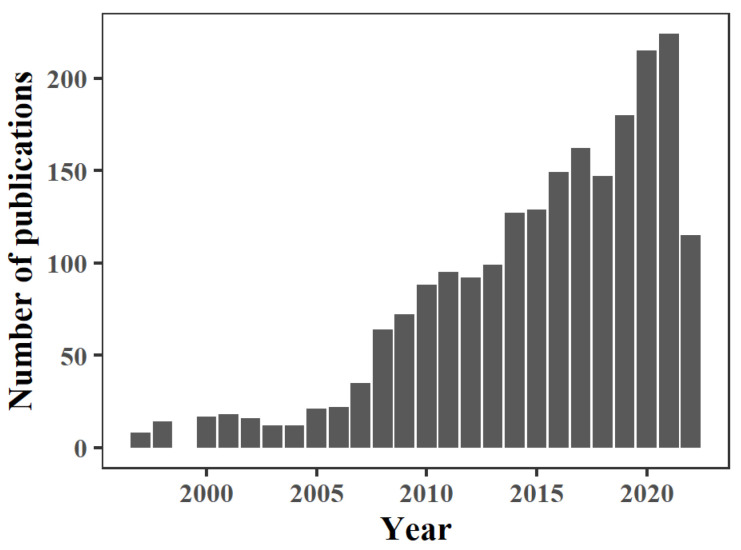
Annual publications on climate change and VBDs. Articles identified by searching the Web of Science with the combination of the following key words: “climate change” and “vector-borne disease”; counts as of 11 August 2022.

As a climate-sensitive type of disease, VBDs are assessed on a global scale with the aim of shedding light on possible future trends, particularly given the increased likelihood of climate change [15]. The impact of climate change on VBDs has become an indisputable fact, and is creating new challenges for public health. As a category, VBDs include rodent-borne (plague, hemorrhagic fever, hemorrhagic fever with renal syndrome, leptospirosis, cutaneous leishmaniasis, and Puumala hantavirus), mosquito-borne (malaria, dengue, Zika, chikungunya, West Nile virus, Ross River virus, and Japanese encephalitis), tick-borne (tick-borne encephalitis, Lyme disease, etc.), and other arthropod-borne diseases [16,17]. Over 700,000 people die from VBDs each year, and more than 80% of the global population lives in high-risk areas threatened by one or more types of VBDs [18]. Of the 250 countries around the world, 86% (218 countries) are suitable for arboviral disease survival and reproduction [19]. Accordingly, a large number of scientists have devoted considerable effort to studying the impact of climate change on VBDs, with 2133 related studies having been published as of August 2022 (Figure 1).

The influence of climate factors on the transmission and spread of VBDs can be considered at the levels of local climate, regional climate, and extreme climate. Local climate, represented by temperature, rainfall, and wind, mainly affects the transmission of VBDs by affecting their vectors [20]; regional climate, represented by the El Niño–Southern Oscillation (ENSO) [21], North Atlantic Oscillation (NAO) [22], Pacific Decadal Oscillation (PDO) [23], and Indian Ocean Dipole (IOD) [24], mainly has indirect impacts on VBDs through affecting local climate [25]. Meanwhile, extreme climate events such as heat waves, cold waves, floods, and droughts increase the risk of VBD spillover [26]. We additionally discuss the interaction between non-climatic factors (e.g., land use and human mobility) and climate factors as relates to VBD transmission and spread [27,28,29]. Moreover, we also discuss the impact of coronavirus disease 2019 (COVID-19) on climate change and the effects of the COVID-19 pandemic on the outbreak risk and incidence of VBDs [30]. All told, our paper aims to comprehensively assess the impacts of climate change on the transmission and spread of VBDs so as to support the precise prevention and control of and comprehensive intervention in VBDs.

## 2. Non-Linear Effects of Local Climate on VBD Transmission

The effects of local climate factors (mainly considering temperature, precipitation, wind) on VBD transmission are generally nonlinear. These factors can affect the distribution range, population dynamics, and virus transmission ability of vectors [29], and hence the developmental response of pathogens [31].

**Figure 2:**
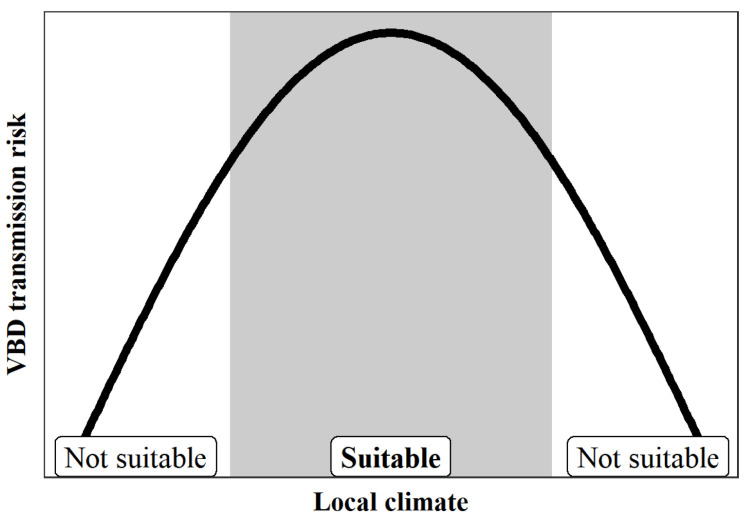
The effect of local temperature on VBD transmission shows a clear n-shape. Suitable temperature promotes the development and transmission ability of VBD vectors, while unsuitable temperature will affect reproduction and mobility and even cause death of vectors.

The non-linear effect of temperature on VBD transmission generally follows an n-shape (Figure 2). Under suitable temperature conditions, the climate adaptability of VBD transmission will be relatively high. When the temperature does not reach suitable conditions, the risk of VBD transmission increases with the increase of temperature, and when the temperature exceeds the peak of the suitable temperature, the risk of VBD transmission decreases with the increase of temperature. Under unsuitable temperature conditions, vector survival may be reduced [32], thereby reducing the transmission capacity of VBDs [33], which also directly affects the development of vector-dependent pathogens [34]. For example, the survival and reproduction range of rodents is generally 10.0–30.0 °C, while 20.0–30.0 °C is the suitable temperature range for rodent-borne disease transmission [35]. The predicted epidemic growth of plague outbreaks is positive between 11.7 °C and 21.5 °C, with a maximum around 17.3 °C [36]. With regard to mosquito-borne diseases, temperature can affect the development and survival of mosquitoes, and there is a thermal optimum which will be suppressed at either heat or cold [37]; including malaria, dengue and Zika, temperature and climate change are reported to have strong nonlinear effects on ectothermic vectors and parasites. The temperature range for the transmission of mosquito-borne diseases is generally 9.0–38.0 °C, with the most suitable range being 23.0–29.0 °C [38].

Precipitation has also demonstrated a nonlinear effect on VBD transmission in many studies. A suitable level of precipitation may be beneficial for the formation of vector breeding habitats [39], especially in deserts, which can provide rich food sources for rodents [40]. Precipitation is also generally beneficial to mosquito oviposition and reproduction, while the relationship between rainfall and incidence of malaria first increases and then decreases with increasing precipitation, reaching its peak at 120 mm [41]. With regard to ticks, a span of more than 28 precipitation days leads the number of ticks to increase significantly [42]; however, large amounts and long periods of precipitation can wash away ticks, their eggs, and their larvae, thus reducing the population [43].

High wind speeds hamper mosquitos in their flight, can decrease the density of mosquitoes, and make them less likely to stand on and bite their hosts. An example to support this view is the finding that high wind impeded the rate of West Nile virus transmission; conversely, there is no obvious negative trend in the effect of low wind speed on mosquitoes. [44,45]. This also represents a non-linear effect on VBD transmission.

Climate change has an important impact on the transmission of vector-borne diseases, which in general will expand the climate-adaptive transmission zone of vector-borne diseases. In Europe, climate change is likely to expand ticks into higher latitudes and altitudes, thereby increasing the incidence of tick-borne diseases [46]. In South Africa, however, rising temperatures could decreases habitat suitability for some tick species (Acari: Ixodidae), which will decrease the occurrence of the related diseases [47]. Under climate scenarios from the IPCC, the climatic suitability of chikungunya transmission will increase in western and central parts of Europe, but will not generally be suitable in Southern Europe [48]. Due to climate change, the suitability of rodents in certain high-altitude areas has increased by 40% [49]. For mosquitoes and ticks, warming climate generally increases the risk of associated disease transmission at high-latitude and -altitude areas, while the risk of transmission may generally decrease in tropical regions. For rodent-borne diseases such as plague, rodents and fleas both influence pathogen transmission; there is uncertainty about the effect of high temperatures on the inhibition of fleas (vectors) and flea-mediated transmission of pathogenic bacteria [50].

## 3. Time-Lag Effect of Local and Regional Climate Impacts on VBD Transmission

**Figure 3:**
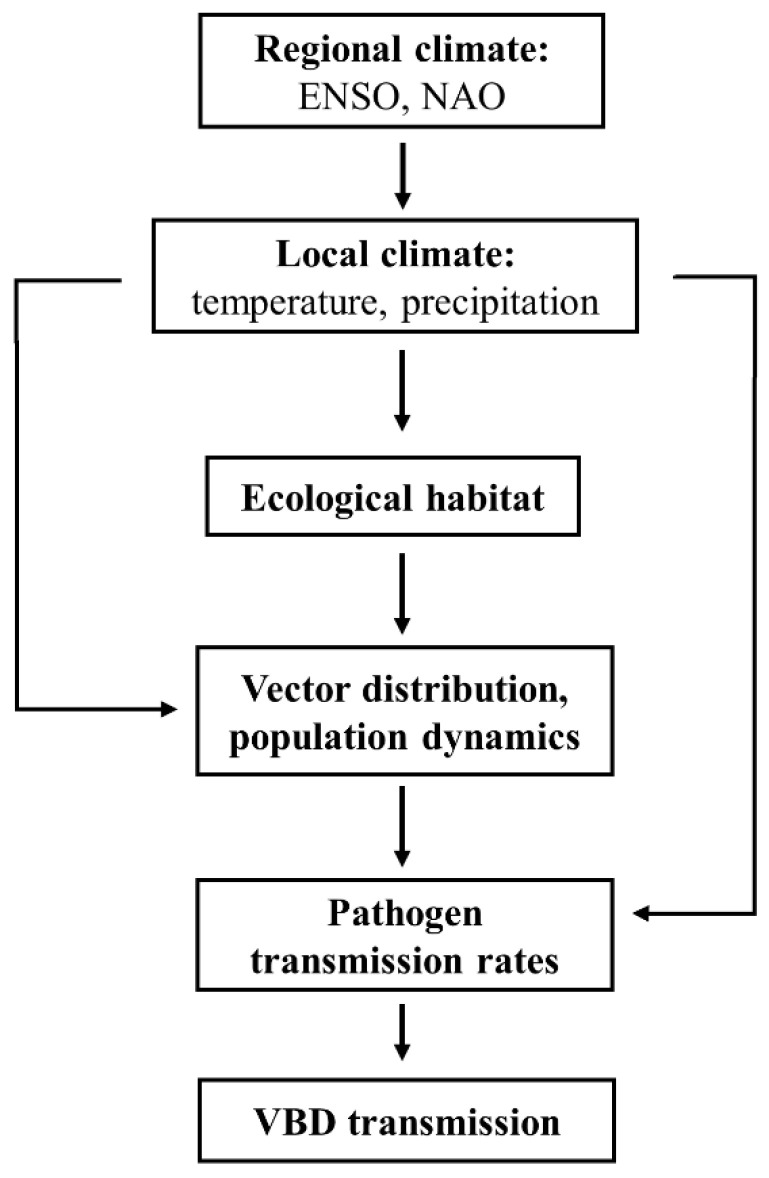
The indirect time-lagged effect of regional climate on VBD transmission in a bottom-up ecosystem. For example, temperature and precipitation have 0–2 month lagged effects on dengue transmission, while the impact of ENSO lags by 3–6 months.

Regional climate mainly exerts its influence on VBDs through local climate factors, which in turn affect the ecological habitat, distribution, and population dynamics of the vectors [51], and hence the transmission rate of the pathogens [24,52], thereby impacting outbreaks of VBDs [53,54,55]. The complexity of these indirect effects can create additional time-lag effects (Figure 3).

Numerous studies have concluded that local climate has a time-lag effect on VBD transmission in the short term. For rodent-borne diseases, many studies have also demonstrated a time-lag effect of regional climate, such as on renal hemorrhagic fever [56,57], leptospirosis [58], and cutaneous leishmaniasis [59]. The time-lag is 1–6 months or even one year due to the complex biological characteristics of rodent-borne diseases [35]; for example, temperature affects the human plague in Arizona and New Mexico with a 2–3 month lag effect, while precipitation has a 1–2 year lag effect [60]. For mosquito-borne diseases, a large number of studies have investigated time-lag effects, including on dengue fever [61,62], malaria [63,64,65], chikungunya [66], Ross River virus [67,68,69], and Japanese encephalitis [70]. The time-lag for these diseases is usually considered to be about 0–2 months due to indirect effects on the life history and density of mosquitoes [71].

Time-lag effects of regional climate have wider ranges and longer timespans than local climate effects, and hence are more relevant to making predictions for disease prevention in advance. ENSO is the most significant example of quasi-periodic climate variability on an interannual scale that can affect weather all over the world [21]. The pattern of global climate variability associated with ENSO has been shown to impact a number of infectious diseases, including rodent-borne diseases [24], mosquito-borne diseases [72,73], and tick-borne diseases [59]. For example, increases in the rate of human plague in China were well-associated with ENSO over short periods (2–3 years), medium periods (6–7 years), and long periods (11–12 years, 30–40 years) [74]. ENSO-driven dengue cases in India between 2010–2017 were likewise positively associated with a 3–6 month time-lag [62], which would help us to predict human outbreaks in advance. However, the ENSO index does not seem to be an accurate index of climate variability in Europe; instead, the NAO has been found to impact outbreaks of 13 infectious diseases [22]. Moreover, multi-decadal temperature changes have been shown to influence the NAO–plague correlation, with 15–22 years lagged impact in different European regions [75]. All told, these regional climates clearly affect the occurrence of VBDs and human health by influencing precipitation and temperature [76,77,78], and could be used as early signals for disease control and prevention.

## 4. Impact of Extreme Climate Distribution Expansion on VBD Spread

**Figure 4:**
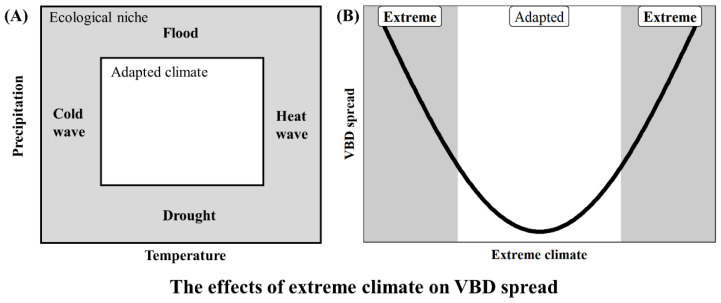
Extreme climate is located at the edge of the ecological niche, and unlike adaptive climate, can affect the spread of VBDs. (**A**) Extreme climate, namely events beyond the range of adaptive climate conditions (heat waves, cold waves, floods, and droughts), has attracted increasing attention as such events can lead to spread of VBDs. (**B**) The u-shaped effect of extreme climate on VBD spread. Climate extremes have a greater risk of spillover than the adaptive climate conditions.

With the frequency of climate extremes increasing as climate change accelerates, it is increasingly important to understand the impact of climate range edges and limitations on VBDs (Figure 4A). Most extreme climate conditions have a u-shaped effect on the spread of VBDs. Under adaptive climatic conditions, the lowest risk of VBD spread is usually observed, whereas under extreme climates which lower or increase the conditions of adaptive climate, the risk of VBD spread will increase. This means that extreme climate events may increase the risk of disease transmission and the spillover of VBDs, whereas under adaptive climatic conditions, the expansion of VBDs is lower (Figure 4B). Extreme climate has been found to be one of the main causes of disease outbreaks and is a cause of alarm in the global community. The impact of climate change on VBDs is more significant in the fringes of different climatic areas, which strongly influences the geographical distribution of vectors [20].

Different types of extreme climate events (heat waves, cold waves, floods, and droughts) have different effects on the distribution expansion of VBDs. The impact of heat waves on mosquitoes depends on the onset time and duration; such events usually promote mosquito population growth in early developmental stages, but often suppress the entire life cycle [79]. Thus, under short-term heat waves, it is advised to guard against the spreading of mosquito-borne VBDs caused by rapid growth of mosquitoes. On the other hand, an experimental study in Kenya found that cold waves during summer months were more favorable for mosquito growth on account of the extremely warm year-round temperature; hence, cold waves in Kenya keep summer cooler and are conducive to VBD spread [80]. Meanwhile, floods wash away the aquatic stage of mosquitoes and their eggs from their breeding sites [81], while the stagnant water left after flood recession provides a suitable habitat for mosquitoes [82,83,84]. When wetlands experience occasional droughts, mosquito populations suddenly explode as their predators and competitors are eliminated [85]. Such increases in mosquito populations would also lead to high-risk spillover of mosquito-borne infectious diseases. Climactic trends also impact VBDs; for example, in northern China (arid climate), rodents are expected to respond positively to high precipitation, whereas in southern China (humid climate), excessive precipitation would destroy rodent nests [86], which impacts human plague intensity due to its positive correlation with rodent density [87].

Thus, in the context of climate change, climate extremes are increasingly expected to create additional risks and possibilities for the spread of VBDs [88,89]. When there is extreme heat in winter, the lack of snow cover makes contact between bank voles and humans easier, such as that which occurred to produce the Puumala hantavirus (PUUV) epidemic of 2006–2007 [90]. An Ecuador study found that under extreme climate, *Aedes aegypti* can expand its distribution in mountainous areas by up to 4215 km^2^, which would put over 12,000 people at risk of disease [37]. In India, an increase of heat wave events has made chikungunya and dengue diseases more prevalent in coastal districts, and Japanese encephalitis and malaria more prevalent in interior districts [91]. Extreme heat, drought, and flooding all have a negative impact on tick distribution, which may disrupt the habitat of *Ixodes* ticks in Europe, especially Northern and Central Europe; however, extreme weather is expected to expand the distribution of *Ixodes* ticks in Europe by 3.8% during 2040–2060, and tick-borne encephalitis (TBE) is expected spread to high altitudes and latitudes [92]. In New York State in America, the annual number of Lyme disease cases increase 4–10% under mild winter temperatures, and increase 2% under extended spring and summer days [93].

## 5. Interaction between Non-Climate and Climate Factors Alters VBD Spread

Beyond climate factors alone, the interactions of non-climatic factors (land use and human mobility) and climate factors are important to consider for their impacts on VBD spread [27,28,94]. 

The interaction of land use and climate change will provide opportunities for pathogen exchange among geographically isolated wildlife, and thus in some cases will promote disease spillover [95]. Projections under climate change and land use in 2070 have indicated that in Asia and Africa, species will converge into new communities at high altitudes, biodiversity hotspots, and areas of high population density, resulting in approximately 4000 times greater cross-species transmission of their associated viruses [95].

Human mobility is also a major factor in the global spread of VBDs [96,97]. With climate change making some areas uninhabitable (as with the severe drought in sub-Saharan Africa), the interaction of climate change and human mobility will manifest as viruses traveling along with mass migrants [27]. 

## 6. The COVID-19 Pandemic Introduces a New Situation for VBD Epidemics

The impact of the COVID-19 pandemic on climate change is debatable in the short term [98,99,100,101]; however, COVID-19 as a background may result in new circumstances that impact the occurrence of VBD epidemics. On the one hand, human activities and air pollution have been reduced during lockdowns in the COVID-19 pandemic, and climate change has been mitigated [98]. On the other hand, a similar reduction in global SO_2_ emissions was found to weaken the aerosol cooling effect, which can lead to warming [102]. These possible climate changes may have new effects on the transmission and spread of climate-sensitive VBDs.

Besides climate change, lack of vector testing and control activities [103,104,105,106] and insufficient financial support for VBD surveillance [107,108] during the COVID-19 pandemic have led to increased prevalence of VBDs. Routine vector testing and control activities required by the Department of Prevention and Control, such as regular household surveys, have been suspended during COVID-19 quarantine [103,104,105,106]. Many countries have temporarily suspended adult surveillance and larval control measures for Aedes aegypti, resulting in an increased risk of dengue transmission [109]. At the same time, due to the economic pressure caused by COVID-19, financial support for VBD surveillance is insufficient [107,108]. In addition, some VBDs with similar symptoms have been marginalized and underdiagnosed during the COVID-19 pandemic, resulting in VBDs being ignored rather than eliminated [30,110].

However, lockdown policies have greatly reduced imported cases and blocked sources of VBD transmission [110,111,112]. Statistics indicate that the number of vector-borne cases declined dramatically during the COVID-19 pandemic in many countries [111,112,113,114]. There are two important reasons for the decrease in the number of VBDs. One reason is that entry–exit control in different countries have greatly reduced imported cases and blocked the source of disease transmission [110]. The other is that the decreasing of human outdoor activities and physical distancing interventions reduced the bite chance of mosquitoes, and consequently reduced the risk of mosquito-borne disease transmission [111,112]. Therefore, prevention and control targeting COVID-19 transmission also has a preventive effect on VBDs.

## 7. Conclusions

In this paper, we summarized the different impacts of multiple climatic factors on the transmission and spread of VBDs in the context of climate change. Local climate exerts non-linear direct effects, resulting in rapid transmission in suitable conditions and decline in an unsuitable environment, with local temperature in particular showing a clear n-shape. Regional climate has an indirect impact on VBDs, affecting transmission through its effects on local climate, which by necessity produces a certain time-lag for the effect on disease transmission. Extreme climate events can increase the spread of disease, leading to the expansion of VBD distributions. Moreover, land use and human mobility have an important interaction effect on VBD spread, increasing the possibilities for spread and spillover. The impact of the COVID-19 pandemic on how climate change affects VBD transmission and spread is yet uncertain.

Quarantine policies during the COVID-19 pandemic successfully blocked the import of VBD cases; this effective prevention and control policy is worth adopting and applying in the field of VBDs. Meanwhile, the impact of COVID-19 on climate change is controversial, and its potential effect on VBDs may gradually become clear in the future. Accordingly, it remains necessary to further explore the potential of COVID-associated climate change to drive effects on VBDs. Meanwhile, the improvement of surveillance systems in relation to the COVID-19 pandemic and the construction of a surveillance network are also worthy of application in VBD surveillance. With the continuous improvement of monitoring systems, it also becomes necessary to adopt methods from the fields of machine learning and artificial intelligence to handle large databases with complex algorithms in the future.

There is still a lot of research worth undertaking with regard to climate change and VBDs. One important research direction is to integrate multidisciplinary factors to analyze the impact of climate change on VBDs, especially with reference to the fields of computer science, zoology, entomology, ecology, and epidemiology. Through the integration of multiple disciplines, we can not only better understand the impacts of climate change on VBDs, but also develop a deeper understanding of the occurrence and development mechanisms of these infectious diseases. Such findings could contribute to achieving a better understanding of how climate change drives effects on VBD risk and spread, thereby improving the prevention and control of VBDs and so improving human health.

## Data Availability

Not applicable.
